# Study the Antimicrobial Resistance and Virulence Factors of Campylobacter Jejuni and Campylobacter Coli Isolated from Poultry Meat: Campylobacter in poultry meat

**DOI:** 10.31661/gmj.v14i.3776

**Published:** 2025-04-16

**Authors:** Hosein Razavian, Leila Golestan, Zohreh Mashak, Mohammad Ahmadi

**Affiliations:** ^1^ Department of Food Hygiene, Ayatollah Amoli Branch, Islamic Azad University, Amol, Iran; ^2^ Department of Food Hygiene, Science and Research Branch, Islamic Azad University, Tehran, Iran; ^3^ Department of Food Hygiene, Karaj Branch, Islamic Azad University, Karaj, Iran

**Keywords:** Campylobacter Species, Antibiotic Resistance, Virulence Factors, Poultry Meat

## Abstract

**Background:**

Poultry meat is recognized as a potential reservoir of Campylobacter jejuni and Campylobacter coli. This study was done to assess antibiotic resistance and virulence characteristics of C. jejuni and C. coli isolated from raw poultry meat.

**Materials and Methods:**

Raw poultry meat samples were collected. C. jejuni and C. coli were isolated after microbial examination. Disk diffusion was applied to apprise antibiotic resistance. Polymerase Chain Reaction was employed to determine the virulence and antibiotic resistance gene distribution.

**Results:**

Raw poultry meat samples contamination rate with Campylobacter spp. was 19% (76 out of 400 samples). The highest contamination rate was observed amongst the raw duck meat samples (37.50%). Forty-three (56.57%) and twenty (26.31%) out of 76 Campylobacter spp. were recognized as C. jejuni and C. coli, respectively. C. jejuni and C. coli isolates harbored the uppermost rates of resistance toward tetracycline (67.44% and 50%), gentamicin (60.46% and 50%), ampicillin (48.89% and 40%), and erythromycin (48.89% and 35%), respectively. The prevalence of multidrug-resistant C. jejuni and C. coli was 81.39% and 75%, respectively. C. jejuni and C. coli bacteria harbored tetO (23.48% and 45%), cmeB (44.18% and 45%), and blaOXA (44.18% and 35%) antibiotic resistance genes, respectively. All isolates harbored fla and ciaB. Among the C. jejuni isolates, cadF (67.44%), racC (46.51%), and cdtB (46.51%) and amongst the C. coli isolates, pldA (50%), cdtA (35%), racC (30%), and cadF (30%) were major virulence factors.

**Conclusion:**

The role of raw poultry meat, particularly duck and goose, as antibiotic-resistant and virulent Campylobacter spp. reservoirs were confirmed.

## Introduction

Campylobacter species are imperative intestinal microbiota of domestic animals, livestock, and poultries. The bacteria have zoonotic aspects and can cause severe foodborne diseases characterized by gastroenteritis, abdominal cramps, diarrhea, vomiting, and even death, named Campylobacteriosis [1][2]. The bacteria can also cause more severe extragastrointestinal diseases, such as Guillain-Barré and irritable bowel syndromes, and arthritis [3]. Nearly 165 million diarrhea and 38,000 deaths annually have been stated for human campylobacteriosis [4]. The economic burden caused by Campylobacteriosis outbreaks and cases of hospitalization and treatment has been estimated to be about 1.5 to 7 billion Dollars in the United States [5].

Poultry provides ideal Campylobacter growth circumstances, as the bird’s bodily temperature is 42°C and Campylobacter spp. growth excellently at 42°C [6]. Consequently, the manipulation and contaminated meat consumption, particularly poultry, are documented as an initial source of human infection [7]. Additionally, epidemiological investigations have revealed that contaminated poultry product consumption is a causative agent for above 80% of Campylobacter cases in the human population [8].

Campylobacter jejuni (C. jejuni) and C. coli are the chief bacteria for the mainstream of human gastroenteritis cases [9][10]. They have several factors responsible for their virulence characteristics, particularly adhesion to host cells, toxin production, and invasion. phospholipase A (pldA), cytolethal distending toxin (cdt), flagellar agent (flaA), Campylobacter fibronectin adhesive factor (cadF), chaperone protein (dnaJ), Campylobacter secretory system IV (virB11), Campylobacter regulatory protein R (racR), Campylobacter invasion antigen B (ciaB), Guillain‐Barré associated genes (wlaN and cgtB), lipoprotein of the enterochelin binding (ceuE) are the most imperative reasons for the C. jejuni and C. coli pathogenesis [11][12].

C. jejuni and C. coli-associated diseases may necessitate antibiotic therapy. Nevertheless, both C. jejuni and C. coli bacteria exhibited high rates of resistance against dissimilar antibiotics, predominantly penicillins, tetracyclines, cephalosporins, beta-lactamase, aminoglycosides, fluoroquinolones, penems, and even macrolides [13][14]. Diverse antibiotic resistance genes are activated in severe cases of antibiotic resistance. Campylobacter spp. antibiotic resistance is mostly arbitrated by the aphA‐3 (aminoglycosides-resistance agent), tetO (tetracyclines-resistance agent), blaOXA (β‐lactams-resistance agent). gyrA (fluroquinolones-resistance agent), and cmeB (multidrug efflux pump agent) factors [14][15].

From food protection, clinical, epidemiological, and microbiological aspects, it is very substantial to determine the role of poultry meat, particularly wild poultry meat like duck, goose, partridge, ostrich, and pheasant (which are consumed less) as sources of antibiotic-resistant and virulence Campylobacter spp. Accordingly, the contemporary work was accomplished to evaluate the prevalence, antibiotic resistance properties, and virulence characters of C. jejuni and C. coli strains isolated from raw duck, goose, chicken, partridge, quail, turkey, ostrich, and pheasant meat samples.

## Materials and Methods

**Figure-1:**
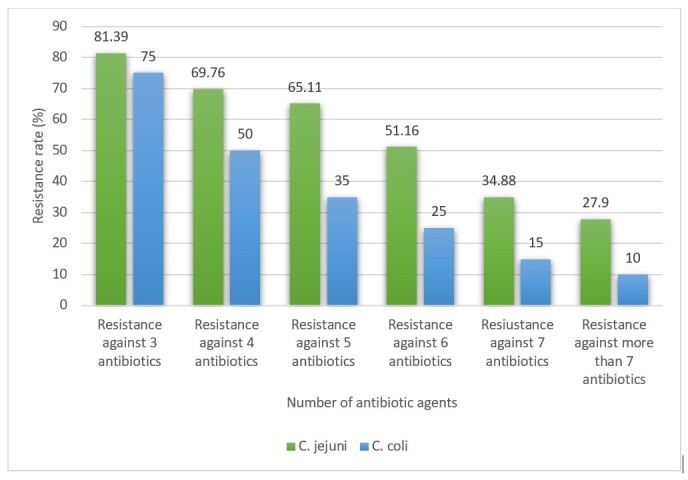
Figure 1. MDR distribution amongst the Campylobacter isolates. The distribution of bacteria that harbored resistance against more than one antimicrobial agent has been added.

**Table:** Table1. Primers, thermal cycles, and PCR ingredients.

Targets	Genes	Sequence (5’-3’)	Size (bp)	Thermal cycles	Ingredients/Volumes (50µL)
	Campylobacter, *16S rRNA *	F: ATC TAA TGG CTT AAC CAT TAA AC R: GGA CGG TAA CTA GTT TAG TAT T	857	**1 cycle:** 10 min: 95°C **35 cycles:** 30 s: 95°C 90 s: 59 °C 1 min: 72 °C **1 cycle:** 10 min: 72 °C	10X PCR buffer: 5 µL Mgcl_2:_1.5 mM dNTP: 200 µM Primer F: 0.5 µM Primer R: 0.5 µM DNA polymerase (Taq): 1.25 U DNA: 2.5 µL
Species identification	*C. jejuni*, *mapA*	F: CTA TTT TAT TTT TGA GTG CTT GTG R: GCT TTA TTT GCC ATT TGT TTT ATT A	589
	*C. coli*, *ceuE*	F: AAT TGA AAA TTG CTC CAA CTA TG R: TGA TTT TAT TAT TTG TAG CAG CG	462
	*tetO*	F: GCG TTT TGT TTA TGT GCG R: ATG GAC AAC CCG ACA GAA G	559	**1 cycle:** 2 min: 95°C **30 cycles:** 30 s: 95°C 1 min: 53 °C (*tetO*) 1 min: 50 °C (*cmeB*) 1 min: 49 °C (*blaOXA*) 1 min: 54 °C (*Apha-3*) 1 min: 55 °C (*gyrA*) 1 min: 72 °C **1 cycle:** 8 min: 72 °C	Similar to above
	*cmeB*	F: AGG CGG TTT TGA AAT GTA TGT T R: TGT GCC GCT GGG AAA AG	444
Antibiotic resistance genes	*blaOXA*	F: AGA GTA TAA TAC AAG CG R: TAG TGA GTT GTC AAG CC	372
	*apha-3*	F: TGC GTA AAA GAT ACG GAA G R: CAA TCA GGC TTG ATC CCC	701
	*gyrA*	F: ATG ATG AGG CAA AAA GAG A R: TAA ACT ATG AGG TGG GAT GT	410
	*fla*	F:AAT AAA AAT GCT CAT AAA AAC AGG TG R:TAC CGA ACC AAT GTC TGC TCT GAT T	855	**1 cycle:** 10 min: 95°C **35 cycles:** 30 s: 95°C 90 s: 55 °C 1 min: 72 °C **1 cycle:** 10 min: 72 °C	Similar to above
	*cdtA*	F:CCT TGT GAT GCA AGC AAT C R:ACA CTC CAT TTG CTT TCT G	370
	*cdtC*	F:CGA TGA GTT AAA ACA AAA AGA TA R:TTG GCA TTA TAG AAA ATA CAG TT	182	**1 cycle:** 10 min: 95°C **35 cycles:** 30 s: 95°C 90 s: 49 °C°C 1 min: 72 °C **1 cycle:** 10 min: 72 °C
Virulence factors	*racR*	F:GAT GAT CCT GAC TTT G R:TCT CCT ATT TTT ACC C	584
	*cadF*	F: TTG AAG GTA ATT TAG ATA TG R: CTA ATA CCT AAA GTT GAA AC	400	**1 cycle:** 5 min: 95°C **32 cycles:** 30 s: 95°C 1 min: 45 °C 1 min: 72 °C **1 cycle:** 8 min: 72 °C
	*cdtB*	F:CAG AAA GCA AAT GGA GTG TT R:AGC TAA AAG CGG TGG AGT AT	620	**1 cycle:** 4 min: 95°C **35 cycles:** 30 s: 95°C 70 s: 51 °C 1 min: 72 °C **1 cycle:** 7 min: 72 °C
	*dnaJ*	F:AAG GCT TTG GCT CAT C R:CTT TTT GTT CAT CGT T	720	**1 cycle:** 5 min: 95°C **32 cycles:** 1 min: 95°C 1 min: 53 °C 1 min: 72 °C **1 cycle:** 8 min: 72 °C
	*virb11*	F:TCT TGT GAG TTG CCT TAC CCC TTT T R:CCT GCG TGT CCT GTG TTA TTT ACC C	494	**1 cycle:** 3 min: 95°C **30 cycles:** 1 min: 95°C 1 min: 48 °C 1 min: 72 °C **1 cycle:** 8 min: 72 °C
	*ciaB*	F:TTT TTA TCA GTC CTT A R:TTT CGG TAT CAT TAG C	986	**1 cycle:** 5 min: 95°C **35 cycles:** 30 s: 95°C 1 min: 42 °C 1 min: 72 °C **1 cycle:** 10 min: 72 °C
	*pldA*	F:AAG CTT ATG CGT TTT T R:TAT AAG GCT TTC TCC A	913	**1 cycle:** 4 min: 95°C **30 cycles:** 1 min: 95°C 90 s: 45 °C 1 min: 72 °C **1 cycle:** 7 min: 72 °C
Virulence factors	*WlaN*	F:TTA AGA GCA AGA TAT GAA GGT G R:CCA TTT GAA TTG ATA TTT TTG	672	**1 cycle:** 4 min: 95°C **30 cycles:** 1 min: 95°C 1 min: 46 °C 1 min: 72 °C **1 cycle:** 8 min: 72 °C
	*ceuE*	F:CCT GCT ACG GTG AAA GTT TTG C R:GAT CTT TTT GTT TTG TGC TGC	793	**1 cycle:** 5 min: 95°C **35 cycles:** 30 s: 95°C 40 s: 48.9 °C 1 min: 72 °C **1 cycle:** 10 min: 72 °C
	*cgtB*	F:TAA GAG CAA GAT ATG AAG GTG R:GCA CAT AGA GAA CGC TAC AA	561	**1 cycle:** 4 min: 95°C **32 cycles:** 30 s: 95°C 90 s: 49.9 °C 1 min: 72 °C **1 cycle:** 7 min: 72 °C

### Ethical statement

This research was only conducted on poultry meat samples and the basic principles of this study were confirmed by the ethical council of the Faculty of Veterinary Medicine, Ayatollah Amoli Branch, Islamic Azad University, Amol, Iran (Ethical code No IR.IAU.AMOL.REC.1403.167).

### Samples

During the winter of 2022, 400 raw poultry meat samples, including quail (60 samples), chicken (60 samples), turkey (60 samples), partridge (50 samples), ostrich (50 samples), pheasant (40 samples), goose (40 samples), and duck (40 samples), were collected from retail centers, Mazandaran province, Iran. From each poultry, 10 g raw meat of the tight muscle was collected using tissue forceps and placed in sterile tubes containing buffered peptone water (30 mL, Merck, Germany) and shaken well. Tubes were suggested to the research center using a portable suggested (4±1 °C) within 1 h of collection.

### Campylobacter isolation and species identification

Tubes containing raw meat samples were centrifuged (4000 rpm, 5 min). The supernatant solution was castoff well. The remaining clot was dissolved in a Preston enrichment broth base (30 mL, HiMedia, India) containing horse blood (5% defibrinated) and an antimicrobial agent (FD042; HiMedia, India). Incubation was done in an environment with microaerophilic circumstances (only 5% O2 and 85% N2, and remaining 10% CO2) (AnaeroPak system (Mitsubish, Japan) for 24 h at 42 ˚C. Formerly, 0.1 mL of the contents were inoculated onto a blood agar base containing the FD 006 supplement of the company (HiMedia, India). Plates were incubated with the same environmental circumstances for 48 h at 42 ˚C. Gray flat circular and non-hemolytic colonies were determined as suspected Campylobacter colonies and subjected to Gram staining and further biochemical tests, together with nalidixic acid resistance, catalase, oxidase, and nitrate reduction. Additionally, species identification was accomplished by the Polymerase Chain Reaction (PCR) (Table-1) [16].

### Antibiotic resistance analysis

To assess the phenotypic characteristics of antibiotic resistance, the broth microdilution method was applied to evaluate the C. jejuni and C. coli minimum inhibitory concentrations (MICs) regarding each antibiotic agent. Rendering the company’s guidelines, commercially accessible Campylobacter Sensititre plates (TREK, UK) were applied. Different classes of antimicrobial agents (μg/ml MIC breakpoint unit, Sigma, St. Louis, MO, United States), including macrolides (azithromycin, ≥8, and erythromycin, ≥32), tetracyclines (tetracycline, ≥16), β-lactams (ampicillin, (≥32), quinolones (nalidixic acid, ≥64, and ciprofloxacin, ≥4), aminoglycosides (gentamicin, ≥4), lincosamides (clindamycin, ≥8 μg), and phenicols (chloramphenicol, ≥32) were evaluated [17][18][19]. Bacteria were cultured in Columbia blood agar and incubated (with similar conditions as mentioned above). A standard concentration of 0.5 McFarland was prepared by transferring some typical col,onies to Mueller-Hinton broth (5 mL). Nearly 104 CFU of achieved suspensions was added to Mueller-Hinton agar containing antimicrobial agents (two-fold dilution). Media were also complemented with sheep blood (5% defibrinated). Plates were incubated in similar conditions (microaerobic atmosphere, 24 h at 42 °C). The test had two positive controls of C. jejuni (ATCC 33560) and C. coli (ATCC 33559) and a negative control of mueller-Hinton broth with Tris/EDTA/Sucrose (TES) and horse blood (lysed). Inhibition zones were assessed rendering the Clinical and Laboratory Standards Institute’s recommendations (CLSI) [17].

### DNA extraction, quality assessment, and encoding genes of virulence and antibiotic resistance

For DNA extraction, isolated bacteria were cultured on Bolton broth (Oxoid, UK) media and incubated at similar temperatures, times, and conditions. DNA extraction kit (Thermo Fisher, Germany) was employed for this purpose. The procedure was performed based on the kit’s instructions. Extracted DNA quality was assessed by gel electrophoresis [20][21]. The extracted DNA’s quantity was assessed by spectrophotometric analysis (NanoDrop device, Thermo Scientific, USA) [22]. All PCR runs were performed using the thermocycler device (Eppendorf, Germany). Table-1 reveals primers, thermal cycles, and PCR ingredients [16][23][24][25]..

### Data analysis

All collected data were added to Excel software. Then, all were transferred to SPSS statistical software version 17 (SPSS Inc., Chicago, Ill., USA) for analysis. Chi-square and Fisher’s exact tests were employed for data analysis. All data were analyzed, their relations were determined, and a P-value < 0.05 was applied as statistically significant [26][27][28].

## Results

**Table:** Table2. Campylobacter distribution amongst the inspected samples.

Samples	N. collected		Campylobacter distribution (%)		
		Campylobacter spp.	*C. jejuni*	*C. coli*	Other species
Chicken	60	14 (23.33)	8 (57.14)	4 (28.57)	2 (14.28)
Quail	60	10 (16.66)	5 (50)	3 (30)	2 (20)
Turkey	60	15 (25)	9 (60)	4 (26.66)	2 (13.33)
Partridge	50	10 (20)	6 (60)	2 (20)	2 (20)
Ostrich	50	-	-	-	-
Pheasant	40	-	-	-	-
Goose	40	12 (30)	7 (58.33)	4 (33.33)	1 (8.33)
Duck	40	15 (37.50)	8 (53.33)	3 (20)	3 (20)
Total	400	76 (19)	43 (56.57) ^*^	20 (26.31) ^*^	12 (15.78) ^*^

The frequency was determined based on a total number of 76 Campylobacter spp. isolates.

**Table:** Table3. Campylobacter antibiotic resistance.

Samples (N. positive)					Antibiotic resistance rate (%)					
		Amp	Nal	E15	C15	G10	Az	Cln	C30	T30
Chicken	*C. jejuni* (8)	5 (62.50)	3 (37.50)	5 (62.50)	3 (37.50)	6 (75)	4 (50)	3 (17.50)	2 (25)	6 (75)
	*C. coli* (4)	2 (50)	1 (25)	2 (50)	1 (25)	2 (50)	1 (25)	1 (25)	1 (25)	2 (50)
Quail	*C. jejuni* (5)	3 (60)	1 (20)	3 (60)	2 (40)	4 (80)	1 (20)	1 (20)	1 (20)	4 (80)
	*C. coli* (3)	1 (33.33)	-	1 (33.33)	1 (33.33)	2 (66.66)	-	-	-	2 (66.66)
Turkey	*C. jejuni* (9)	6 (66.66)	4 (44.44)	6 (66.66)	3 (33.33)	7 (77.77)	3 (33.33)	2 (22.22)	3 (33.33)	8 (88.88)
	*C. coli* (4)	2 (50)	1 (25)	2 (50)	1 (25)	3 (75)	1 (25)	1 (25)	-	3 (75)
Partridge	*C. jejuni* (6)	3 (50)	2 (33.33)	3 (50)	3 (50)	4 (66.66)	1 (16.66)	2 (33.33)	1 (16.66)	5 (83.33)
	*C. coli* (2)	1 (50)	-	1 (50)	-	1 (50)	-	-	-	1 (50)
Goose	*C. jejuni* (7)	2 (28.57)	1 (14.28)	2 (28.57)	-	3 (42.85)	1 (14.28)	-	-	3 (42.85)
	*C. coli* (4)	1 (25)	-	1 (25)	-	1 (25)	-	-	-	1 (25)
Duck	*C. jejuni* (8)	2 (25)	1 (12.50)	2 (25)	-	2 (25)	-	-	-	3 (37.50)
	*C. coli* (3)	1 (33.33)	-	-	-	1 (33.33)	-	-	-	1 (33.33)
Total	*C. jejuni* (43)	21 (48.89)	13 (30.23)	21 (48.89)	11 (25.58)	26 (60.46)	10 (23.25)	8 (18.60)	7 (16.27)	29 (67.44)
	*C. coli* (20)	8 (40)	2 (10)	7 (35)	3 (15)	10 (50)	2 (10)	2 (10)	1 (5)	10 (50)

^*^Ampicillin, nalidixic acid, erythromycin, ciprofloxacin, gentamicin, azithromycin, clindamycin, chloramphenicol, tetracycline.

**Table:** Table4. Campylobacter antibiotic resistance genes profile.

Samples (N. positive)				Antibiotic resistance genes (%)		
		*tetO*	*cmeB*	*blaOXA*	*apha3*	*gyrA*
Chicken	*C. jejuni* (8)	5 (62.50)	4 (50)	4 (50)	3 (37.50)	4 (50)
	*C. coli* (4)	2 (50)	2 (50)	1 (25)	1 (25)	2 (50)
Quail	*C. jejuni* (5)	3 (60)	2 (40)	3 (60)	1 (20)	2 (40)
	*C. coli* (3)	1 (33.33)	1 (33.33)	2 (66.66)	1 (33.33)	1 (33.33)
Turkey	*C. jejuni* (9)	5 (55.55)	4 (44.44)	4 (44.44)	3 (33.33)	3 (33.33)
	*C. coli* (4)	2 (50)	2 (50)	1 (25)	1 (25)	1 (25)
Partridge	*C. jejuni* (6)	3 (50)	3 (50)	3 (50)	1 (16.66)	2 (33.33)
	*C. coli* (2)	1 (50)	1 (50)	1 (50)	-	1 (50)
Goose	*C. jejuni* (7)	3 (42.85)	3 (42.85)	2 (28.57)	1 (14.28)	2 (28.57)
	*C. coli* (4)	2 (50)	2 (50)	1 (25)	1 (25)	1 (25)
Duck	*C. jejuni* (8)	4 (50)	3 (37.50)	3 (37.50)	2 (25)	3 (37.50)
	*C. coli* (3)	1 (33.33)	1 (33.33)	1 (33.33)	-	1 (33.33)
Total	*C. jejuni* (43)	23 (53.48)	19 (44.18)	19 (44.18)	11 (25.58)	16 (37.20)
*C. coli* (20)	9 (45)	9 (45)	7 (35)	4 (20)	7 (35)

### Campylobacter contamination rate

Table-2 reveals the Campylobacter contamination rate amongst the inspected samples. The poultry meat contamination rate with Campylobacter spp. was 19% (76 out of 100 samples). The applied method failed to detect any Campylobacter spp. amongst the ostrich and pheasant samples. Raw duck meat samples harbored the maximum contamination rate of Campylobacter spp. (37.50%), even though raw quail meat samples harbored the minimum (16.66%). C. jejuni and C. coli distribution amongst the isolates was 56.57% and 26.31%, respectively. Twelve out of 76 (15.78%) Campylobacter spp. were determined as species other than C. coli and C. jejuni. Data analysis revealed a significant difference between raw poultry meat species and Campylobacter contamination rate (P < 0.05).

### Antibiotic resistance

Table-3 reveals the Campylobacter antibiotic resistance. Isolates of C. jejuni revealed the topmost resistance rate against tetracycline (67.44%), gentamicin (60.46%), ampicillin (48.89%), and erythromycin (48.89%). Isolates of C. coli revealed the topmost resistance rate against tetracycline (50%), gentamicin (50%), ampicillin (40%), and erythromycin (35%). Strains isolated from the sources of duck and goose were less resistant to evaluated agents (P < 0.05). C. coli isolates almost exhibited a lower resistance rate (P < 0.05). A considerable variance was gotten amid the sample type and Campylobacter resistance (P < 0.05).

### Antibiotic resistance genes

Table-4 reveals the Campylobacter antibiotic resistance gene profiles. TetO (23.48%), cmeB (44.18%), and blaOXA (44.18%) were more frequent amongst the C. jejuni strains. TetO (45%), cmeB (45%), blaOXA (35%), and gyrA (35%) were more frequent amongst the C. coli strains. Strains isolated from the sources of duck and goose harbored the lower genes encode antibiotic resistance (P < 0.05). C. coli isolates almost exhibited a lower distribution of antibiotic resistance genes (P < 0.05). Considerable difference was obtained between sample type and genes encode antibiotic resistance distribution (P < 0.05).

### MDR profile

MDR isolates were determined as those harbored simultaneous resistance against at least 3 antibiotic agents. Figure-1 reveals the MDR distribution amongst the Campylobacter isolates. No less than, 81.39% of C. jejuni and 75% of C. coli isolates were strongminded as MDR. The frequency of C. jejuni and C. coli strains with resistance against more than 7 antibiotic agents was 27.90% and 10%, respectively.

### Virulence characters

Table-5 reveals the virulence characteristics of the Campylobacter isolates. All isolates harbored fla (100%) and ciaB (100%) virulence factors. Reversely, none of C. coli isolates harbor dnaJ, virB11, and wlaN virulence factors. CadF (67.44%), racC (46.51%), and cdtB (46.51%) were the most predominant factors amongst the C. jejuni. PldA (50%), cdtA (35%), racC (30%), and cadF (30%) the most predominant factors amongst the C. coli. Evaluated virulence factors were less predominant amongst the C. coli isolates (P < 0.05). Considerable difference was obtained between sample type and virulence factors distribution (P < 0.05).

## Discussion

**Table:** Table5. Virulence characteristics of the Campylobacter isolates.

Samples (N. positive)							Virulence factors (%)							
		*fla*	*cdtA*	*cdtB*	*cdtC*	*racC*	*cadF*	*dnaJ*	*virB11*	*ciaB*	*pldA*	*wlaN*	*ceuE*	*cgtB*
Chicken	*C. jejuni* (8)	8 (100)	4 (50)	5 (62.50)	2 (25)	4 (50)	6 (75)	3 (37.50)	2 (25)	8 (100)	2 (25)	2 (25)	2 (25)	2 (25)
	*C. coli* (4)	4 (100)	2 (50)	1 (25)	1 (25)	1 (25)	2 (50)	-	-	4 (100)	1 (25)	-	-	1 (25)
Quail	*C. jejuni* (5)	5 (100)	3 (60)	2 (40)	1 (20)	2 (40)	3 (60)	1 (20)	2 (40)	5 (100)	1 (20)	1 (20)	-	2 (40)
	*C. coli* (3)	3 (100)	1 (33.33)	1 (33.33)	-	1 (33.33)	1 (33.33)	-	-	3 (100)	2 (66.66)	-	-	1 (33.33)
Turkey	*C. jejuni* (9)	9 (100)	3 (33.33)	4 (44.44)	1 (11.11)	4 (44.44)	6 (66.66)	2 (22.22)	3 (33.33)	9 (100)	3 (33.33)	2 (22.22)	2 (22.22)	1 (11.11)
	*C. coli* (4)	4 (100)	1 (25)	-	1 (25)	1 (25)	1 (25)	-	-	4 (100)	2 (50)	-	-	1 (25)
Partridge	*C. jejuni* (6)	6 (100)	1 (16.66)	2 (33.33)	1 (16.66)	3 (50)	4 (66.66)	2 (33.33)	2 (33.33)	6 (100)	2 (33.33)	1 (16.66)	-	2 (33.33)
	*C. coli* (2)	2 (100)	1 (50)	-	-	1 (50)	-	-	-	2 (100)	1 (50)	-	-	-
Goose	*C. jejuni* (7)	7 (100)	2 (28.57)	3 (42.85)	1 (14.28)	3 (42.85)	4 (57.14)	2 (28.57)	2 (28.57)	7 (100)	2 (28.57)	1 (14.28)	2 (28.57)	2 (28.57)
	*C. coli* (4)	4 (100)	1 (25)	1 (25)	1 (25)	1 (25)	1 (25)	-	-	4 (100)	2 (50)	-	-	1 (25)
Duck	*C. jejuni* (8)	8 (100)	3 (37.50)	4 (50)	1 (12.50)	4 (50)	6 (75)	3 (37.50)	2 (25)	8 (100)	2 (25)	1 (12.50)	1 (12.50)	2 (25)
	*C. coli* (3)	3 (100)	1 (33.33)	1 (33.33)	-	1 (33.33)	1 (33.33)	-	-	3 (100)	2 (66.66)	-	1 (33.33)	-
Total	*C. jejuni* (43)	43 (100)	16 (37.20)	20 (46.51)	7 (16.27)	20 (46.51)	29 (67.44)	13 (30.23)	13 (30.23)	43 (100)	12 (27.90)	8 (18.60)	7 (16.27)	11 (25.58)
*C. coli* (20)	20 (100)	7 (35)	4 (20)	3 (15)	6 (30)	6 (30)	-	-	20 (100)	10 (50)	-	1 (5)	4 (20)

Undercooked or raw poultry meat is recognized as a high-risk food product [29][30]. However, Campylobacter spp. is recognized as the most important foodborne pathogen transferred from undercooked or raw poultry meat to the human population [31]. In the present research, 19% of raw poultry meat samples were contaminated with Campylobacter spp. C. jejuni and C. coli contamination rates amongst the evaluated samples were 10.75% (43/400) and 5% (20/400), respectively. In comparison with surveys conducted in this field [32][33], we reported a lower contamination rate of poultry meat samples. Campylobacter contamination rate amongst chicken meat in Iran [34], chicken meat in west Africa [35], broiler meat in the USA [36], raw turkey meat in Poland [37], ostrich meat in South Africa [38], quail meat in Italy [39], duck meat in South Korea [40], and goose meat in Iran [41] were 28.90%, 32.80%, 25.40%, 49.30%, 24.63%, 21.40%, 77.50%, and 26.10%, respectively.

Our findings showed that both C. coli and C. jejuni bacteria had the maximum contamination rates in raw duck and goose meat samples. This finding can probably be due to the different habitats and diets of these two species. Ducks and geese usually live in wetlands, swamps, and wet areas near rivers and lakes. These areas are probably more polluted with bacteria. Also, the diet of these species is completely different. A higher prevalence of contamination of raw duck and goose meat samples with Campylobacter spp. was also reported in South Korea [40], Iran [41][42], United Kingdome [43], the United States [44], and New Zealand [45]. Hadiyan et al. (2022) [46] reported that the total prevalence of Campylobacter spp, amongst the raw chicken, turkey, Quebec, goose, and ostrich meat samples was 61.66%, 23.63%, 3.07%, 1.53%, and 5.33%, respectively. They also reported that the total C. jejuni and C. coli prevalence were 57.44% and 48.14%, respectively. Similarly, Mousavinafchi et al. (2022) [47] described that the contamination rates of raw chicken, turkey, quail, and goose meat samples with C. jejuni and C. coli bacteria were 30.76% and 5.76%, 9.85%, and 7.04%, 0%, and 8.57%, and 12.50% and 0%, respectively. Sabzmeydani et al. (2020) [48] also reported that the Campylobacter contamination rate of poultry meat was 44.75%, considering the higher prevalence in coot (78.26%), goose (83.33%), duck (84%), chicken (67.78%), and pheasant (66.66%). Our discoveries also exposed that C. jejuni had an advanced contamination rate than C. coli. This discovery was similar to those described by Walker et al. (2019) [49] (Australia) and Mohamed (2019) [50] (Egypt). Studies on pheasant raw meat samples as sources of Campylobacter spp. are scarce in the world. Only 7 papers were available on this matter and they reported the Campylobacter contamination rates of pheasant meat samples between 9% to 70.20% [51][52][53][54][55][56][57][58]. It seems that wild birds, especially geese and duck are more prospective to be accused of transmitting the Campylobacter spp. Wild species can be permanent Campylobacter reservoirs and transmit bacteria to humans as well as domesticated birds. As a result, making decisions to prevent their unsanitary sale seems to be necessary. This matter may also need additional studies on the wild birds role in the Campylobacter transmission to other poultries, animals, and humans.

Isolated Campylobacter harbored significant resistance against common antibiotic agents, particularly tetracycline, gentamicin, ampicillin, and erythromycin. Phenotypic resistance of isolated Campylobacter spp. was assisted with the genotypic distribution of diverse antibiotic resistance genes, particularly tetO, cmeB, blaOXA, and gyrA. Phenotypic and genotypic resistance of Campylobacter spp. was also accompanied by the high distribution of MDR (75 to 81.3% based on the genus of bacteria). These three findings may show an extremely high prescription of antimicrobials in Iran. The Campylobacter strains with wild birds sources (goose and duck) harbored a lower resistance rate. The reason for this finding was the lack of cultivation by humans and as a result, the lack of antibiotic prescription in wild birds. Anadvanced antimicrobial administration in chicken, quail, and turkey is a conceivable cause of the higher antibiotic resistance. Similar to our findings, surveys conducted in Iraq [57], Slovenia [58], Switzerland [59], and Benin [60], specified the boosted Campylobacter resistance against tetracycline, gentamicin, amoxicillin/clavulanic acid, and erythromycin. Rahimi and Ameri (2011) [61] stated that the prevalence of resistance against tetracycline (70.60%), nalidixic acid (54%), and ciprofloxacin (49.70%) was higher amongst Campylobacter with source of poultry meat. Hadiyan et al. (2020) [46] mentioned the diverse resistance rates of C. jejuni against gentamicin (1.85%), ciprofloxacin (33.33%), nalidixic acid (22.22%), tetracycline (31.48%), ampicillin (33.88%), amoxicillin (14.81%), erythromycin (42.59%), azithromycin (20.37%), clindamycin (24.07%), and chloramphenicol (31.48%). Similarly, Casalino et al. (2022) [62] showed that resistance rate of Campylobacter spp. or wild bird origins against azithromycin, erythromycin, chloramphenicol, ciprofloxacin, enrofloxacin, nalidixic acid, tetracycline, gentamicin, and trimethoprim/sulfamethoxazole were 5.90%, 2%, 0%, 45.10%, 31.40%, 23.50%, 17.60%, 0%, and 52.90%, respectively. Alike Campylobacter resistance rates were also labelled in inquiries directed at Poland [63], China [64], Malaysia [65], Latvia [66], and Iran [67]. TetO, cmeB, blaOXA, and gyrA genes which encode resistance against tetracyclines, multidrug efflux pumps, beta-lactams, and fluoroquinolones were also predominant in previous research [46][47]. A survey in Tunisia [68], described that cmeB, tetO, and blaOXA-61 distribution amongst the C. jejuni and C. coli isolates were 80% and 100%, 100% and 80%, and 81% and 93%, respectively. Gharbi et al. (2022) [69] reported that tetO and cmeB were detected in all Campylobacter isolates, while blaOXA-61 was only detected in 18.82% of C. jeuni and 6.25% of C. coli isolates. Hull et al. (2021) [70] indicated the high distribution of blaOXA, aadE1, cmeB, tet(O), and aph amongst the Campylobacter spp. in the United States. Du et al. (2018) [71] also reported the high frequency of aadE (58.90%), tet(O) (98%), aadE‐sat4‐aphA (6.60%), and ermB (20.50%) antibiotic resistance genes amongst the Campylobacter spp. in China. As phenotypically and genotypically majority of isolates harbored resistance toward tetracycline, gentamicin, ciprofloxacin, and beta-lactams, they would not be a suitable candidate for campylobacteriosis treatment.

The presence of antibiotic resistance genes is one of the ways that bacterial strains develop the antimicrobial resistance toward antibiotics. In fact, several other ways such as changes in the cell wall permeability, enzymatic degradation of antibacterial drugs, and alteration of bacterial proteins that are antimicrobial targets, are more important than presence or absence of antibiotic resistance genes. This is the main reason for the low distribution of antibiotic resistance genes among bacteria in this study.

Campylobacter spp. also harbored diverse virulence factors, particularly fla, ciaB, cadF, racC, cdtB, and pldA virulence factors. They are mainly involved in Campylobacter pathogenesis, including bacterial motility (flaA), adhesion of host tissue (dnaJ, cadF, and racR), cytotoxin producing agents (cdt complex), lipoprotein encoding agents (ceuE), Guillain-Barré syndrome occurrence (cgtB and wlaN), and invasive agents (ciaB, virB11, and pldA) [46]. As all isolates harbored fla and ciaB factors, they can easily have motility and invasion of host cells. Considering the high distribution of examined virulence factors, consumption of raw or undercooked poultry meat samples definitely can mediate Campylobacteriosis and subsequent complications in the human population. Scarce investigations have also been performed in this field. Fani et al. (2019) [34] described the distribution of cdtC, cdtB, cdtA, cadF, pldA, and cgtB virulence factors amongst the Campylobacter spp. strains were 100%, 100%, 100%, 100%, 65.40%, and 15.40%, respectively. Hadiyan et al. (2022) [46] also showed the high frequency of ciaB (100%), flaA (100%), dnaJ (81.48%), racR (83.33%), cdtC (79.62%), cdtB (81.48%), and cadF (74.07%) in C. jejuni and also high frequency of ciaB (100%), flaA (100%), cadF (61.53%), and pldA (65.38%) in C. coli isolates. Correspondingly, Gharbi et al. (2022) [68] reported that all Campylobacter isolates harbored cdt (A, B, and C) virulence factors. Furthermore, flaA was detected in 96-100% of Campylobacter bacteria. Moreover, cadF, racR, virB11, pldA, and dnaJ were detected in 89-95%, 78-93%, 89-94%, 79-89%, and 50-71% of Campylobacter isolates [68]. Detected virulence factors have a high portion in the pathogenesis of infections produced by the Campylobacter spp. [11][72]. Consequently, our isolates may be virulent sufficient to reason Campylobacteriosis in people who consume raw or undercooked poultry meat.

This study was limited to the lack of molecular typing of bacterial isolates and also the absence of other poultry samples to monitor the presence of Campylobacter spp.

## Conclusion

In conclusion, C. jejuni and C. coli strains were detected in 10.75% and 5% of raw poultry meat samples, a higher bacterial prevalence in goose (30%) and duck (37.50%). Bacterial distribution is accompanied by a boosted resistance against tetracycline, gentamicin, ampicillin, and erythromycin. Genotypically resistance was attended by a boosted tetO, cmeB, blaOXA, and gyrA antibiotic resistance genes distribution. Isolates also harbored diverse virulence factors, especially fla, ciaB, cadF, racC, cdtB, and pldA. The findings may highlight the raw poultry meat portion, especially wild bird meat samples, as sources of virulent and antibiotic-resistant Campylobacter. Rendering the high prevalence of resistance toward tetracyclines, beta-lactams, and fluoroquinolones which cooperated with a boosted antibiotic resistance encoding genes distribution, their prescription cannot be efficient in Campylobacteriosis. It appears that the contaminated poultry meat consumption containing resistant and virulent C. jejuni and C. coli strains may cause unembellished foodborne diseases and resist routine antimicrobial therapies. Thus, certain alternative antimicrobial agents should be considered for virulent and resistant strains. Alternative sources of antimicrobial materials, particularly with natural bases may prevent the expansion of antimicrobial resistance among the bacteria.

## Conflicts of Interest

The authors have no conflict of interest to declare in regard to this publication.
